# A Couch for a Patient with a Fractured Femur

**Published:** 1861-09

**Authors:** Elmer Nichols

**Affiliations:** Medical Student


### A COUCH FOR A PATIENT WITH A FRACTURED FEMUR.

By ELMER NICHOLS, Medical Student.

As mobility is said to be the most frequent cause of ununited fractures, and having been so unfortunate as to have a fractured femur myself, and knowing the great difficulty in keeping a patient sufficiently quiet with one, I have been induced to contribute my mite to the vast amount that has been written on this subject by giving a description of a couch I invented for the use of patients with a fracture of this kind. It is with a feeling of great responsibility I approach a subject that has engaged the minds of so many eminent surgeons, and one which good teachers treat so carefully, and one, also, which surgeons in general understand none too well, as the deformed limbs we so often meet clearly indicate.

I hope no person will suppose I am so self-elated as to imagine for one moment that I am able to obviate all the difficulties that arise in the treatment of fractured femurs; far from it. If I am able, to some extent, to overcome any one obstacle, I shall feel well compensated for the time I have devoted to the subject. Whether I do this or not depends on the faithfulness in the trial of the following described apparatus:

Make a common lounge frame, as follows: Take two pieces of scantling, the diameters being three and two inches, and six or more feet in length. These unite by *four* cross-pieces of the same diameters and two and a half feet in length. Two of these are placed near the ends, while the other two are placed near the middle of the frame, so that when a person is lying on the lounge the nates will rest over one, while the other will be under the upper portion of the thighs, being about six inches from each other. These cross-pieces, groove on their inner and upper edges for the purpose of holding the

*boards* that form the bottom of the couch. The peculiar position of these slats or boards is this: the ones on which the lower extremities rest are to be even with the top of the frame, extending from the cross-piece at the foot to the next one in the middle, while the others extend from the other middle cross-piece to the one at the head, being situated some lower than the edge of the frame, so as to leave room for a mattrass, and should have a joint near the middle, by which the head and shoulders may be raised or lowered to suit the patient. These need two supports under the middle of these boards—the one at the foot to keep the boards from sagging and producing a curve in the fractured bone, and the one at the head to support the hinge-joint. There are two light cross pieces mortised into the longitudinal pieces. Between the two middle cross-pieces are placed two short pieces of boards, filling up the space between them, except an aperture on the medium line through which are passed the fœces. Suspended beneath this is a board on which is placed a vessel at the time the bowels are evacuated.

A firm mattrass is placed over the upper portion of this frame, and secured throughout to the boards by means of strings passing through it. This mattrass must be firm and unyielding, giving way only enough to fill up unevenness of the body, and when completely depressed be on a level with the top of the frame and boards on which the fractured limb rests. There should be an excavation in the cross-piece on which the nates rest, and this should be well padded by the mattrass, so as not to press too hard on them. The whole couch, except the orifice through which the fœces pass, is covered with a quilt and sheet, or anything similar to keep the patient warm, and thick enough to be somewhat soft; but not depressable to any great extent.

The longitudinal pieces may extend beyond the frame at each end and be converted into handles, making a sort of hand-barrow on which the patient may be moved. Thus when a patient receives the injury away from home, as is often the case, he can easily be placed on the couch, taken into a sled or large wagon, and by two persons, one at the head and the other at the feet, steadying it in their hands over rough roads, he may be conveyed home, even a long distance, though it may be, without material injury. When where the patient wishes to remain, the frame can rest on two wooden horses or benches.

*The Treatment with this Apparatus.*—This will be as I should treat it myself without reference to any particular rules laid down in books on fractured femurs, though I believe it is not contrary to the general plan.

Having placed the patient on the couch, you put a long, straight splint beneath the limb, padding it sufficiently to fill up inequalities of the limb.

Having placed the fractured ends of bone in apposition, you apply the eighteen-tailed bandage. On the outer side of the limb place the long splint, extending from the hip to below the foot, and on the rest of the thigh apply short, thin, but firm splints, extending from the groin to the knee-joint. These are held in position by single, strong bands, tied snugly around the whole. Padding, of course, is put beneath the splints so as to obtain pressure as the thigh requires. Now, if the fracture is oblique it is necessary to apply extension to keep the limb in proper length. This may be accomplished in two ways: First, by the usual means, with the long splint, with a shoe adjusted by means of a screw to draw it out or let it back as you wish, while the upper end of the splint is held in place by means of the spiral band, filled with lint or cotton, passing round the groin and through the holes in the splint. This, probably, is as good as any. There is but one objection, and that is the shoe, or the mode in which the straps are often applied, produces excoriation if you use the desired force for extension. This, however, can be obviated by using a legging instead of a shoe, which is to pass up over the calf of the leg. This is to be tightly wound with a bandage, and then saturated with rosin dissolved in alcohol.

The legging and bandage should be previously rubbed with powdered rosin. But better than this, perhaps, is the use of long, wide straps of adhesive plaster, extending all the way up the leg and over the knee-joint.

The other mode is perhaps the most readily procured, especially on the couch. The spiral band passes around the groin and extends up opposite the waist, is made fast to the side of the couch. A shoe, with legging or two straps extending up the leg, (as described above,) with two eyes in the sole of it, through which passes two cords back to a hook or peg in the foot-piece. The object of the *two* eyes (one near the head and the other near the toe) is to keep the foot at right angles with the leg. The cords running back from these eyes are tied as snugly as possible to the hook or peg in the foot-piece, and now, by placing a stick between the cords and twisting all necessary extension will be obtained. To prevent the foot from turning from side to side, a cord is tied round the toe of the shoe and fastened to each side of the couch.

To prevent the patient moving his hips, and thus tending to move the fractured bone, a broad belt passes over them and is buckled on each side to straps tacked on the frame. Care should be had not to press the pelvic viscera with the belt.

*What I Claim in This as Superior to Other Dressings.*— I understand they have beds constructed in large hospitals for patients with fractured limbs, having a trap-door through which the fæces can pass. If the bed is hard and firm, I conceive this to be as good, and perhaps better, than the couch above described; but it is not available to the common practicing surgeon, while the couch may be constructed after the fracture has been dressed, and then the patient placed apon it as easily as he could be raised for the purpose of evacuating the bowels; and it will *pay* to have it prepared for *only one* case of fractured femur, *i. e.*, if a *good limb* is better than a poor, crooked excuse for one.

There have been many means devised for raising the patient from the bed, when necessary, by means of rollers, levers, &c. But I dislike the idea of moving the patient at all, let him be ever so careful to obey every injunction of the surgeon, and besides have the most careful attendants, (which, be assured, you will hardly ever find,) there will be continually occurring sundry jars and twistings that will cause more or less gliding of the fractured ends on each other, thus retarding union. The bed on which the patient must of a necessity be placed is usually yielding, allowing of depression, thus allowing the patient to glide about, moving the limb, and thus requiring an excessively tight dressing to prevent curvature to a *great extent.* On the couch the patient has for his limb and body a plane, unyielding surface, and is kept in *one place all the time*, so that it requires but a very little pressure in the dressing to keep the fractured ends of bone in perfect apposition; and this I deem to be an important point to be gained, as I believe a bone would unite quicker if the circulation was not impeded at all by any dressing, if the limb could be kept *perfectly quiet.*

Since I commenced this article I have been shown a sort of barrow, made by tacking a piece of canvass to a wooden frame, on which the patient lies, and is raised on and off the bed when necessary. This has the objections, first, of the sagging of the canvass, causing movement and curvature; second, *moving the patient;* and third, requiring two attendants, who are liable to have accidents occur, even if they are the most careful. In mine, one attendant is all that is required at any time after the limb is once dressed.

The arrangement for raising the patient's head and shoulders is one to be desired, as it is one the patient will wish for, and wishing, *will have it*, spite of all injunctions to the contrary. If there is no means provided he will resort to pillows, chairs, &c., which will cause more or less jarring and movement of the body, and through that the same to the fractured bone, in placing them in position.

Now, when we look at the great number of suits brought against surgeons for maltreatment of this fracture, I feel sure that this article will not simply be glanced at unheeded; but that the apparatus herein described will be thoroughly tested, and its merits (if it has any) be made known, as there is no patent on it and is free to all who will accept it. When you have a case of fractured femur, and are puzzled to know what to do, refer your mind to the couch prepared by one who has had this unfortunate fracture himself.

---

PODOPHYLLINE.—I have been in almost daily practice of using it for several years, and have made up my mind respecting its potent qualities. It undoubtedly does act as a stimulant to the liver, which, under its influence, pours out bile profusely. This action is not temporary, but is persistent for several days after its administration. It is, therefore, an admirable adjuvant to other cathartics which are apt to be followed by constipation, requiring a subsequent laxative to again move the bowels three or four days after their exhibition. Given alone, it is apt to gripe, and it should therefore be administered with some laxative, as jalap, or rhubarb and some carminative. Care should be taken in its administration to children, for I have known it to be so excessive in its action as to produce something approaching collapse. Another peculiarity in its action is, that it is very persistent, continuing to operate for several days upon the intestinal canal; being in this respect the opposite of most medicines, which are apt to be followed by a greater or less constipation proportionate to the catharsis.—*Prof. Gardner.*

